# UPLC-ESI-MS/MS-based widely targeted metabolomics reveals differences in metabolite composition among four *Ganoderma* species

**DOI:** 10.3389/fnut.2024.1335538

**Published:** 2024-03-18

**Authors:** Liu Wei-Ye, Guo Hong-Bo, Yang Rui-Heng, Xu Ai-Guo, Zhao Jia-Chen, Yang Zhao-Qian, Han Wen-Jun, Yu Xiao-Dan

**Affiliations:** ^1^College of Biological Science and Technology, Shenyang Agricultural University, Shenyang, China; ^2^College of Life Engineering, Shenyang Institute of Technology, Fushun, China; ^3^Institute of Edible Fungi, Shanghai Academy of Agricultural Sciences, Shanghai, China; ^4^Alpine Fungarium, Tibet Plateau Institute of Biology, Lasa, China

**Keywords:** widely targeted metabolomics, *Ganoderma*, *Ganoderma lingzhi*, *Ganoderma sinense*, *Ganoderma leucocontextum*, *Ganoderma tsugae*

## Abstract

The Chinese name “Lingzhi” refers to *Ganoderma* genus, which are increasingly used in the food and medical industries. *Ganoderma* species are often used interchangeably since the differences in their composition are not known. To find compositional metabolite differences among *Ganoderma* species, we conducted a widely targeted metabolomics analysis of four commonly used edible and medicinal *Ganoderma* species based on ultra performance liquid chromatography-electrospray ionization-tandem mass spectrometry. Through pairwise comparisons, we identified 575–764 significant differential metabolites among the species, most of which exhibited large fold differences. We screened and analyzed the composition and functionality of the advantageous metabolites in each species. *Ganoderma lingzhi* advantageous metabolites were mostly related to amino acids and derivatives, as well as terpenes, *G. sinense* to terpenes, and *G. leucocontextum* and *G. tsugae* to nucleotides and derivatives, alkaloids, and lipids. Network pharmacological analysis showed that SRC, GAPDH, TNF, and AKT1 were the key targets of high-degree advantage metabolites among the four *Ganoderma* species. Analysis of Gene Ontology and Kyoto Encyclopedia of Genes and Genomes demonstrated that the advantage metabolites in the four *Ganoderma* species may regulate and participate in signaling pathways associated with diverse cancers, Alzheimer’s disease, and diabetes. Our findings contribute to more targeted development of *Ganoderma* products in the food and medical industries.

## 1 Introduction

“Lingzhi” is the Chinese term for a certain type of edible and medicinal fungi with a long history, generally including *Ganoderma lingzhi*, *Ganoderma sinense*, *Ganoderma tsugae*, and *Ganoderma leucocontextum* (1–3). *Ganoderma* contains a diverse range of bioactive compounds, including polysaccharides, triterpenoids, polyphenols, nucleotides, amino acids, alkaloids, and vitamins (4–6). These active compounds exert remarkable pharmacological effects and are therefore intensively researched and widely applied in the medical field (7). The pharmacological properties of *Ganoderma* have been demonstrated in clinical trials and therapeutic applications, encompassing its anticancer, antioxidant, immunomodulatory, hypoglycemic, cardioprotective, anti-inflammatory, antiviral, and neuroprotective effects (8–11).

*Ganoderma* is also highly favored in the food industry (12). For thousands of years, it has been utilized as a health-promoting food supplement renowned for its properties of relaxation, mental clarity, and physical well-being (13, 14). Currently, *Ganoderma* is primarily marketed in health products and nutritional supplements, such as teas, alcoholic beverages, beverages, capsules, oral solutions (12, 14). *Ganoderma* and derived products represent a multibillion-dollar industry worldwide (15). Thus, *Ganoderma* has great potential in the food and medical industries.

However, there remain some prominent and challenging issues in the development of the *Ganoderma* industry. The most significant issue is that it is currently uncertain whether there are differences in the composition and contents of active components among different species. If so, it may be possible to develop different *Ganoderma* products based on the advantages of each species in future, which would be of great importance for the further development of *Ganoderma* for the food and medical industries. Widely targeted metabolomics tools are suitable for addressing the aforementioned issues as they offer high throughput, high sensitivity, and high qualitative accuracy, and comprehensive databases are available (16).

In this study, we collected samples of the four commonly cultivated *Ganoderma* species: *G. lingzhi*, *G. sinense*, *G. tsugae*, and *G. leucocontextum* (17). We analyzed the composition of the active components in each species and identified significantly differential metabolites using widely targeted metabolomics based on ultra performance liquid chromatography-electrospray ionization-tandem mass spectrometry (UPLC-ESI-MS/MS). The research findings are beneficial for the development of *Ganoderma* in the food and medical industries.

## 2 Materials and methods

### 2.1 Sampling and sample extraction

The abbreviations used for the fungal species in this manuscript and the production areas of the four *Ganoderma* species are presented in Table 1. The fruiting bodies of all *Ganoderma* species are harvested upon reaching maturity through artificial cultivation. The criteria for determining *Ganoderma* fruiting body maturity are disappearance of the white growth ring of the fungus cap, cessation of expansion of the fungus cap and continuous thickening, and browning of the edge of the fungus cap (24, 25). Three replicate samples (1 g) from fungal caps were collected for each species. Using vacuum freeze-drying technology, place the biological samples in a lyophilizer (Scientz-100F), then grinding (30 Hz, 1.5 min) the samples to powder form by using a grinder (MM 400, Retsch). Next, weigh 50 mg of sample powder using an electronic balance (MS105DM) and add 1200 μL of −20°C pre-cooled 70% methanolic aqueous internal standard extract (less than 50 mg added at the rate of 1200 μL extractant per 50 mg sample). Vortex once every 30 min for 30 sec, for a total of 6 times. After centrifugation (rotation speed 12000 rpm, 3 min), the supernatant was aspirated, and the sample was filtered through a microporous membrane (0.22 μm pore size) and stored in the injection vial for UPLC-MS/MS analysis.

**Table 1:** Sample details of the four *Ganoderma* species.

Sample abbreviation	Species name	Production area	Host plant
Gl	*Ganoderma lingzhi*	Changbai Mountain, Jilin province, China	*Castanea, Cyclobalanopsis* and *Quercus* (18)
Gs	*Ganoderma sinense*		*Albizia mollis, Quercus* sp., *Dendrocalamus strictus* and *Dipterocarpus* sp. (19, 20)
Gt	*Ganoderma tsugae*		*Larix* sp., *Picea* sp. and *Tsuga* sp. (20–22)
Gz	*Ganoderma leucocontextum*	Linzhi City, Tibet province, China	*Cyclobalanopsis glauca* (20, 23)

### 2.2 UPLC conditions

The sample extracts were analyzed using an UPLC-ESI-MS/MS system (UPLC, ExionLC^™^ AD, https://sciex.com.cn/; MS, Applied Biosystems 6,500 Q TRAP, https://sciex.com.cn/). The analytical column was Agilent SB-C18 (1.8 μm, 2.1 mm × 100 mm), and the mobile phase comprised solvent A, pure water with 0.1% formic acid, and solvent B, acetonitrile with 0.1% formic acid. Sample measurements were performed with a gradient program comprising 95% A, 5% B, linear gradient to 5% A and 95% B within 9 min, 5% A and 95% B for 1 min, 95% A and 5.0% B within 1.1 min and kept for 2.9 min. The flow rate was 0.35 mL/min, column oven temperature was 40°C, and the injection volume was 2 μL. The effluent was alternatively connected to an ESI-triple quadrupole (QQQ)-linear ion trap (QTRAP)-MS.

### 2.3 ESI-q trap-MS/MS

The ESI source operation parameters were as follows: source temperature, 500°C; ion spray voltage (IS), 5,500 V (positive ion mode)/−4,500 V (negative ion mode); ion source gas I (GSI), gas II (GSII), curtain gas were 50, 60, and 25 psi, respectively; and collision-activated dissociation, high. QQQ scans were acquired under multiple reaction monitoring (MRM), with collision gas (nitrogen) set to medium. Declustering potential (DP) and collision energy (CE) analyses for individual MRM transitions were performed, with further DP and CE optimization. A specific set of MRM transitions was monitored for each period according to the metabolites eluted within this period.

### 2.4 Qualitative and quantitative analyses of metabolites

Crucial MS parameters, such as DP, CE, retention time (RT), Q1 (precursor ion), and Q3 (product ion), were used to identify metabolites from the Metware database (Wuhan Metware Biotechnology Co., Ltd.). After identifying the compounds, we conducted a comparative analysis against the database and classified them into Class I and Class II based on their structural characteristics. Class I represents the primary classification of compounds, while Class II provides a more detailed categorization of metabolites in Class I. Metabolites were quantified using the MRM mode for mass spectrum peaks of metabolites.

### 2.5 Reconstruction and analysis of protein–protein interaction networks

The canonical SMILES of *Ganoderma* metabolites were retrieved from the PubChem and NPASS databases. Subsequently, protein targets for each compound were predicted using SwissTargetPrediction, with a restriction to *Homo sapiens*. The identified targets of each dominant *Ganoderma* metabolite were merged, and duplicates were eliminated. PPI networks for all targets were predicted using information provided by the STRING database, with target genes restricted to “*Homo sapiens*” genetic symbols and saved as tsv files. The tsv files were visualized in Cytoscape v3.9.1 to depict the PPI network. To identify the key targets, we conducted target screening within the PPI network of all targets. The screening criteria were as follows: key targets were determined based on a degree value greater than twice the median, and betweenness centrality, and closeness centrality values exceeding the median. The selected key targets were subsequently visualized using STRING and Cytoscape v3.9.1, leading to the construction of a PPI network for each key target of each *Ganoderma* metabolite.

### 2.6 Gene ontology functional annotation and Kyoto encyclopedia of genes and genomes pathway analysis

To elucidate the biological processes and signaling transduction roles of key target proteins, we employed the ClueGO tool (a Cytoscape plug-in) for GO functional annotation and KEGG pathway analysis. The analysis was performed by inputting a list of human target gene names. The pathways wherein genes were located were filtered, retaining only GO items with *p* values <0.01, while ensuring that each pathway contains more than 20 genes and has a ratio higher than 20%. The top 20 pathways were selected based on their ratio, and a bubble plot visualization was generated using the ggplot2 package in R.^1^

### 2.7 Statistical analysis

Unsupervised principal component analysis (PCA) was performed using the statistics function prcomp in R. The data were unit variance-scaled before unsupervised PCA. Pearson correlation coefficients between two samples were calculated using the cor function in R and the data are presented as heatmaps. In differential metabolite analysis, differential metabolites between two groups were determined based on a value importance plot (VIP) value >1 and |Log_2_fold change (FC)| ≥ 1.0. VIP values were extracted from orthogonal projections to latent structures-discriminant analysis (OPLS-DA) results, which also contain score plots and permutation plots, generated using the R package MetaboAnalystR. The data were log-transformed and mean-centered before OPLS-DA. To avoid overfitting, a permutation test (200 permutations) was used.

## 3 Results and discussion

### 3.1 Metabolite composition of the four *Ganoderma* species

Widely targeted metabolomics analysis revealed the presence of 1,187 metabolites in the Gl, Gs, Gz, and Gt samples. All detected metabolites were categorized into 11 classes under Class I: amino acids and derivatives (387), lipids (140), alkaloids (119), organic acids (110), others (110), phenolic acids (92), terpenoids (91), nucleotides and derivatives (84), flavonoids (36), lignans and coumarins (10), and quinones (8). Polysaccharides and triterpenoids have been the focus of research on the pharmacological activities of *Ganoderma* (26, 27). The fruiting body is the most important pharmacological part of *Ganoderma*. Xie et al. (6) demonstrated that the fruiting body exhibited the highest abundance of metabolites compared to the fermentation broth, mycelium, and spores of *G. lucidum*. Additionally, most triterpenoids were exclusively detected in the fruiting body, thereby establishing the fruiting body as a more promising candidate for the development of anti-tumor and anti-AIDS drugs (6). However, the chemical composition of *Ganoderma* is remarkably complex, and efforts are underway to broaden the research scope of its pharmacological constituents (28, 29). We found that amino acids and their derivatives were the most abundant metabolites in each *Ganoderma* species, providing new insights for the development of *Ganoderma* products. Amino acids and derivatives play diverse specific physiological roles in human life activities, and derived products represent an established market globally (30–32). Our findings indicate that *Ganoderma* is suitable for developing amino acid-based health products, such as nutritional supplements and beverages. In addition, *Ganoderma* is rich in other functional components such as alkaloids and organic acids, but research on their composition and functionality is limited. Based on the detected metabolites, we analyzed the metabolite composition of each *Ganoderma* species.

### 3.2 Differential metabolite composition of each *Ganoderma* species

**Figure 1:**
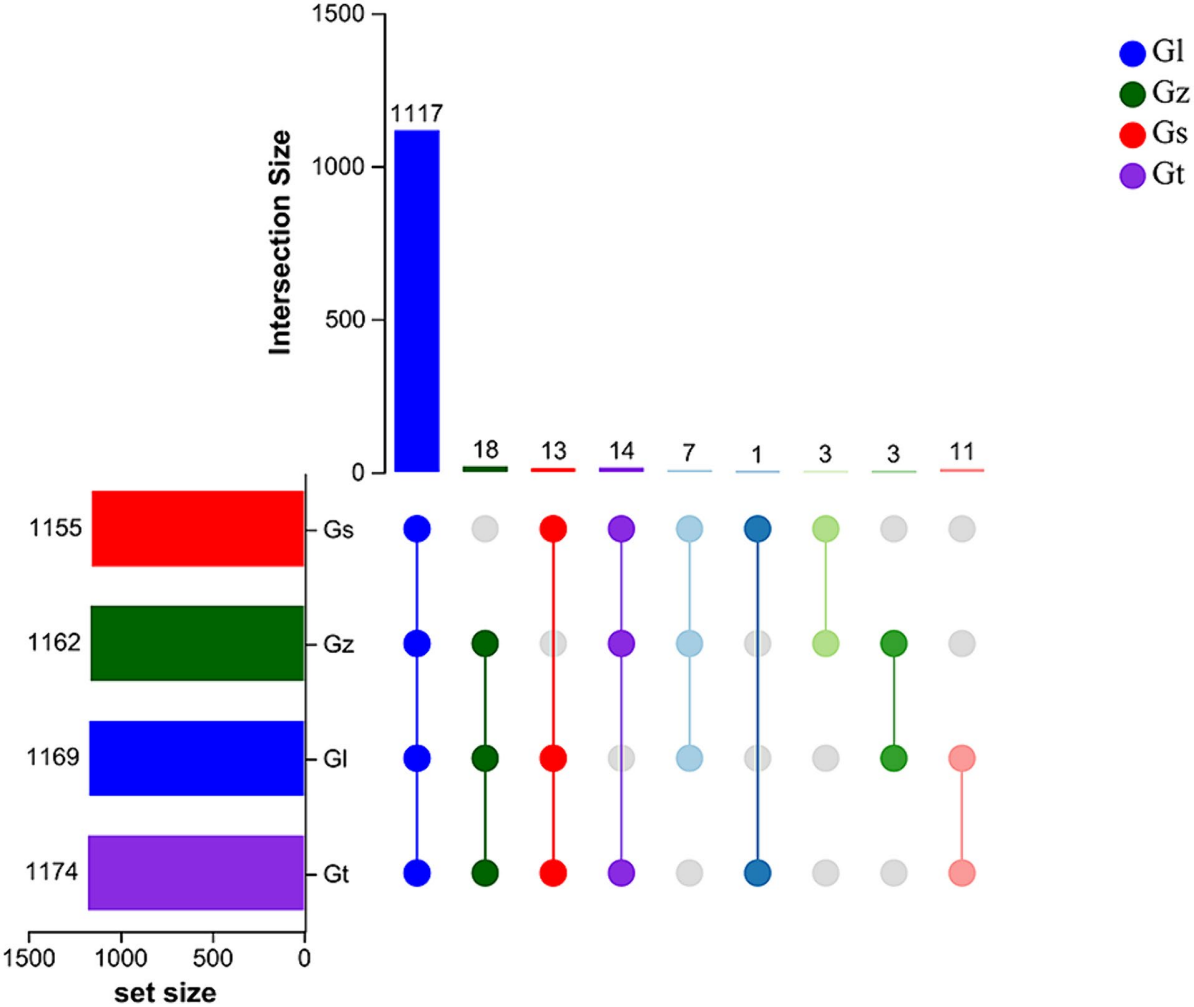
Upset Venn plots of metabolite numbers in the four *Ganoderma* species.

The upset Venn diagram in Figure 1 shows the numbers of metabolites in each species. We detected a total of 1,187 metabolites, including 1,117 common metabolites, in all four species, with Gt having the widest variety of metabolites (1,174), followed by Gl (1,169), Gz (1,162), and Gs (1,155). Thus, there were only minor differences in metabolite variety among the four *Ganoderma* species were.

**Figure 2:**
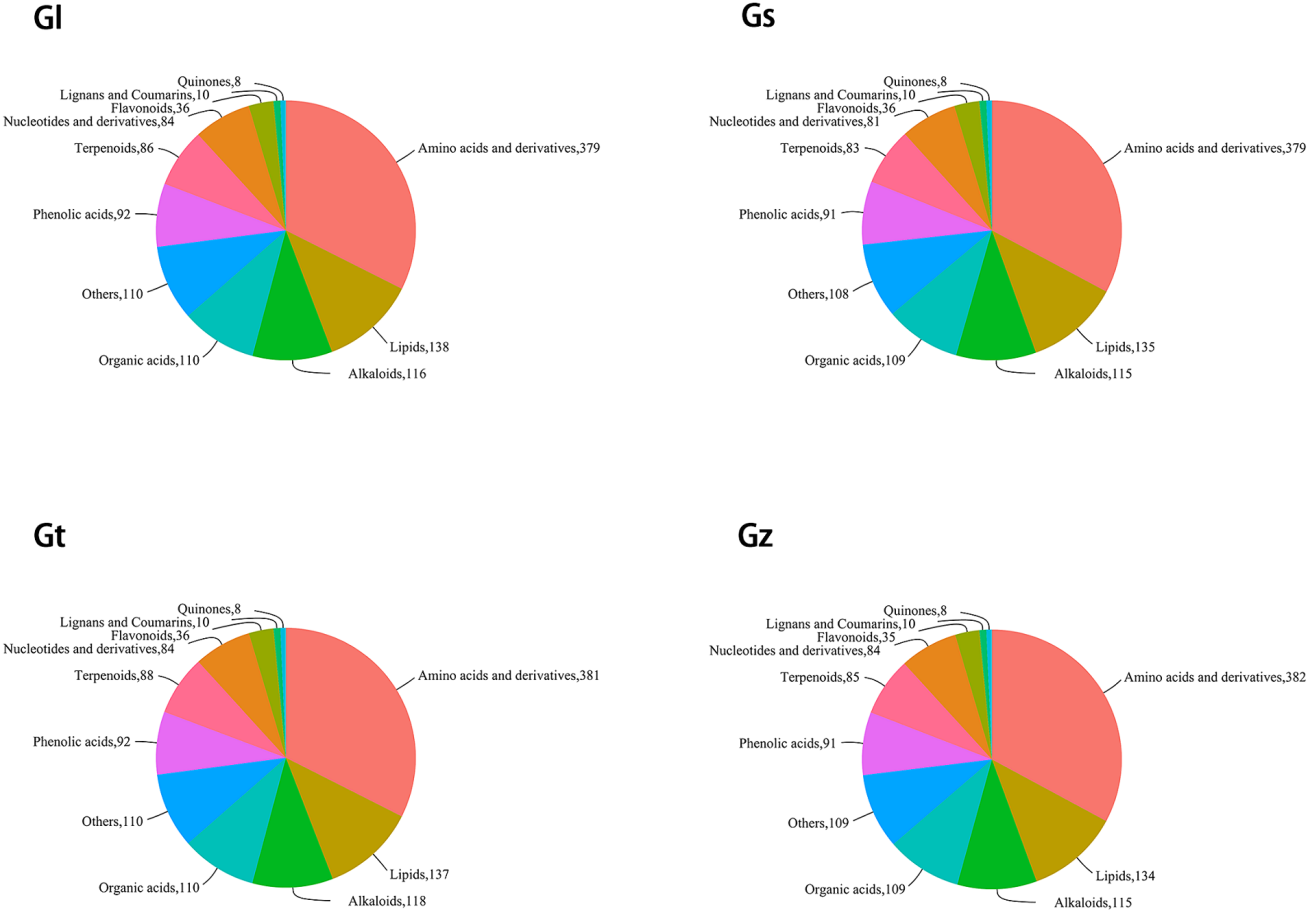
Pie chart of classification of metabolites in each *Ganoderma* species.

The pie chart in Figure 2 shows the numbers of metabolites in the different categories in each *Ganoderma* species. Among all species, Gz was the most abundant in amino acids and derivatives, Gl in lipids, and Gt in alkaloids and terpenoids. Differences in the numbers of metabolites in other categories were relatively small (≤3).

**Figure 3:**
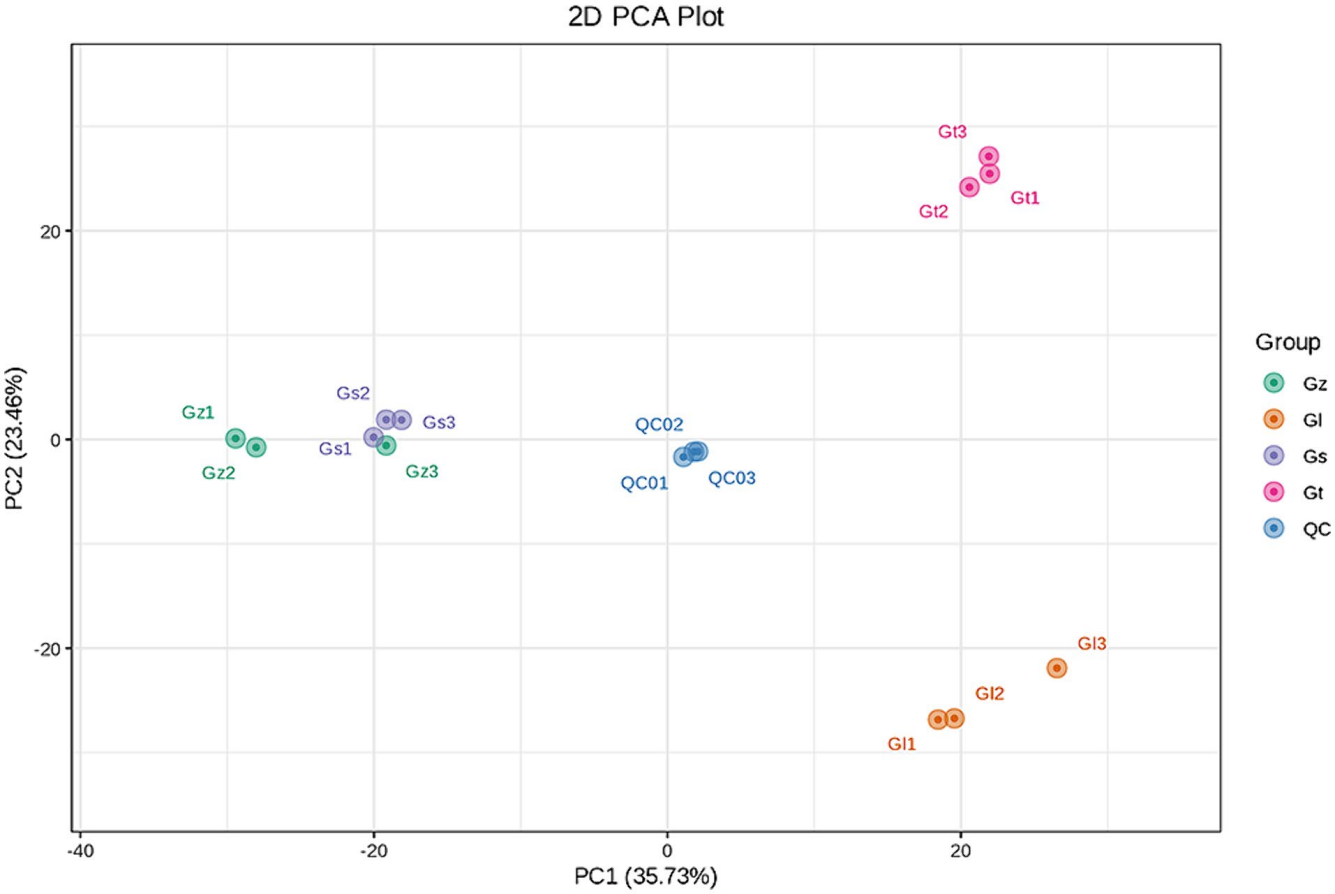
PCA score plot for the four *Ganoderma* species.

The interrelationships in metabolite composition among all *Ganoderma* species were investigated using PCA (Figure 3). PC1 and PC2 contributed 35.73 and 23.46%, respectively, of the metabolic variation among all species. The central sample Qc was a mixture of equal amounts of all samples and served as a quality control. The three samples from each *Ganoderma* species clustered closely together, indicating good reproducibility of the triplicate measurements. The distance between Gz and Gs was short, whereas Gl and Gt were clustered further away from the other species. These findings indicated significant differences in metabolite composition among the different *Ganoderma* species.

### 3.3 Selection and analysis of advantageous metabolites in each *Ganoderma* species

#### 3.3.1 OPLS-DA

To maximize the differentiation among groups and screen differential metabolites, an OPLS-DA model was established using multidimensional statistics (33). The results indicated that differential metabolites could be screened based on VIP values (Supplementary Figure S1).

#### 3.3.2 Differential metabolite analysis among the four *Ganoderma* species

**Figure 4:**
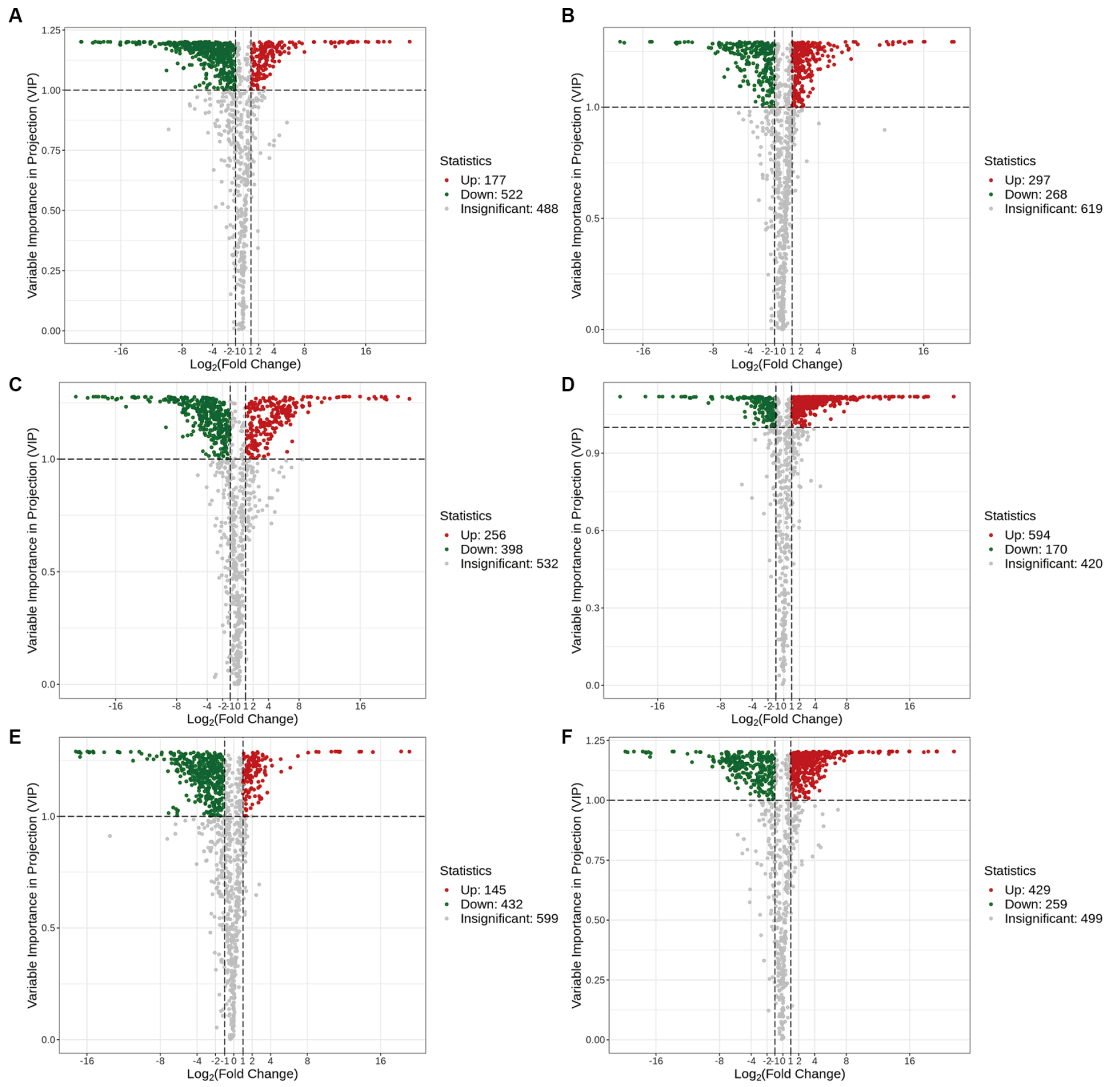
Volcano plots of the differential metabolites in the four *Ganoderma* species. **(A)** Gl vs. Gs; **(B)** Gl vs. Gt; **(C)** Gl vs. Gz; **(D)** Gs vs. Gt; **(E)** Gz vs. Gs; **(F)** Gz vs. Gt.

**Figure 5:**
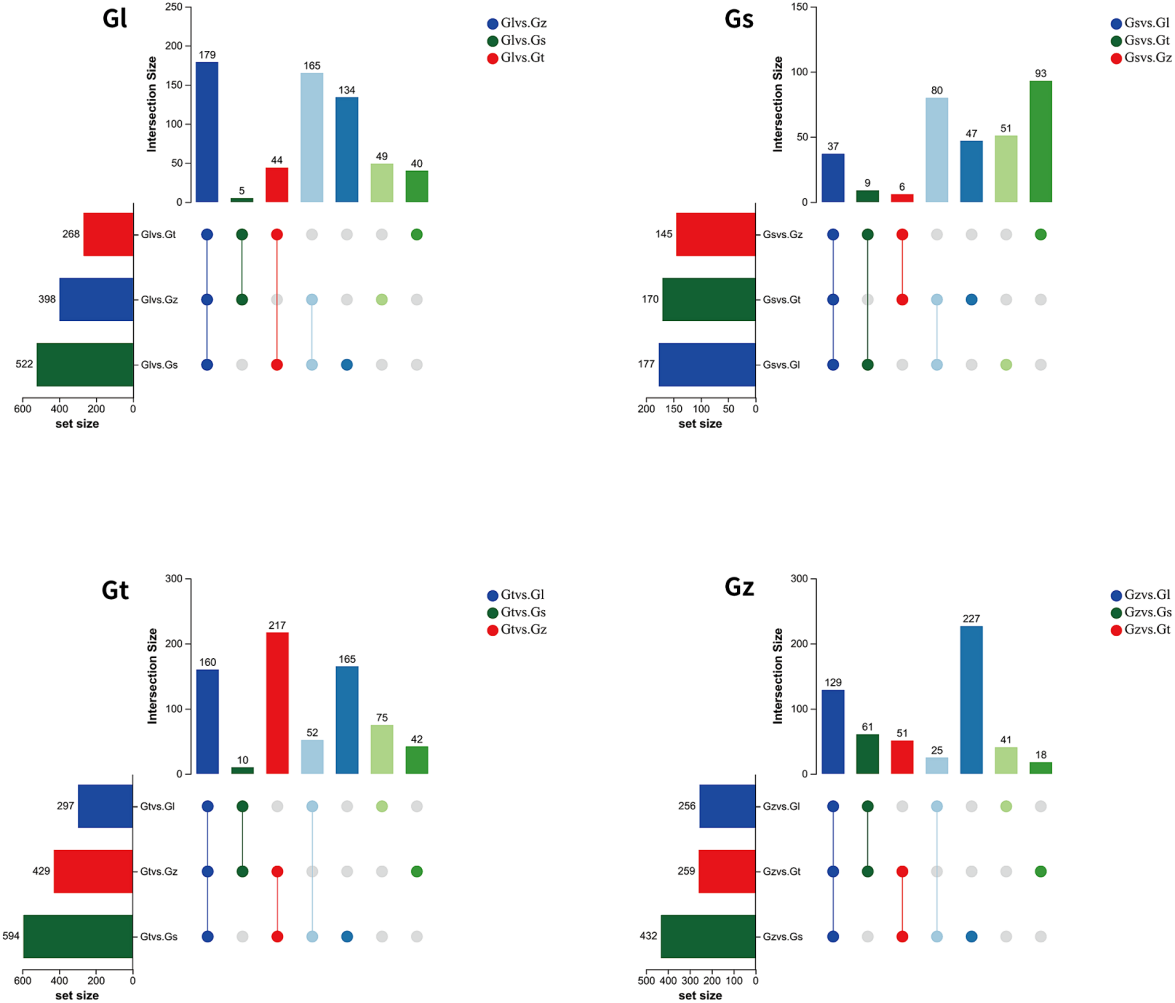
Upset Venn diagrams of metabolites specifically enriched in each of the four *Ganoderma* species.

The volcano plots in Figure 4 show the numbers of differential metabolites (both significantly and insignificantly) in pairwise comparisons of all *Ganoderma* species. There most significantly differential metabolites (764) were found between Gs and Gt, and the least (565) between Gl and Gt (Table 2). The metabolites that were significantly more abundant in one species than in the other three were regarded advantageous metabolites and are represented in an upset Venn diagram (Figure 5). According to the numbers of advantageous metabolites, the species were ranked in the following order: Gl (179) > Gt (160) > Gz (129) > Gs (37).

**Table 2:** Numbers of significant and non-significant differential metabolites among the four *Ganoderma* species.

Group name	Significant	Down regulated	Up regulated	Insignificant
Gl vs. Gs	699	522	177	488
Gl vs. Gt	565	268	297	619
Gl vs. Gz	654	398	256	532
Gs vs. Gt	764	170	594	420
Gz vs. Gs	577	432	145	599
Gz vs. Gt	688	259	429	499

#### 3.3.3 FCs in advantageous metabolites among *Ganoderma* species

**Figure 6:**
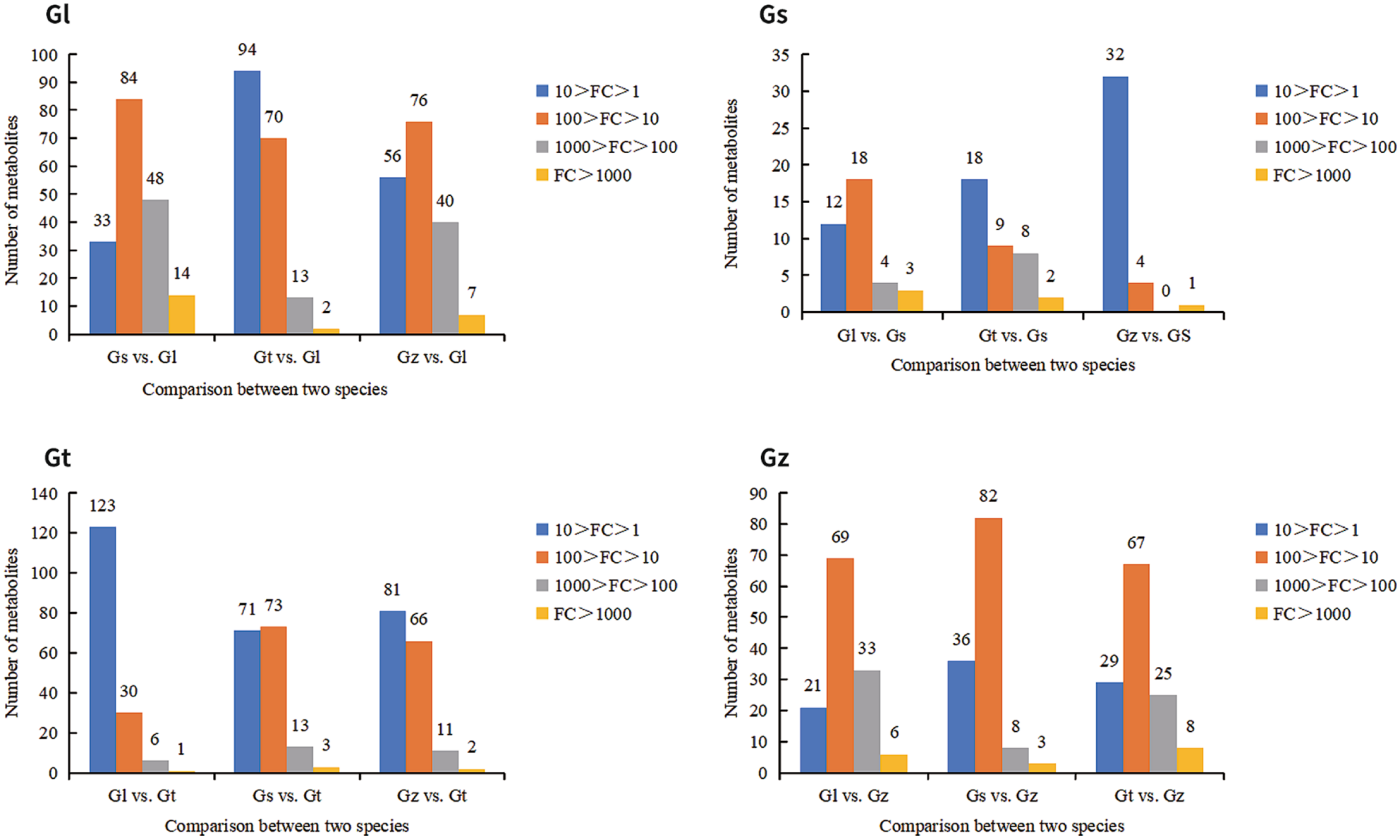
Number of advantage metabolites of each *Ganoderma* species in different FC intervals.

FCs represent differences in the relative abundance of metabolites. We statistically analyzed the FC of advantageous metabolites to evaluate their level of dominance (Figure 6). In general, the majority of advantageous metabolites in Gl and Gz were found in the larger FC range, whereas the majority of predominant metabolites in Gt and Gs showed smaller FCs. The advantageous metabolites with FC > 1,000 are listed in Supplementary Table S1. Most of these metabolites were unique to one species, as observed in pairwise comparisons. Some metabolites, e.g., terpenoids in Gs, exhibited a FC > 1,000-fold between two species.

### 3.4 Composition of advantageous metabolites of different categories among all *Ganoderma* species

Advantageous metabolites reflect the characteristics in which one *Ganoderma* species excels over the others. Therefore, we will discuss the advantageous metabolites of each species based on the classifications of the compounds. The pie charts in Supplementary Figures S2, S3 show the composition of advantageous metabolites in Classes I and II of each species.

Within Class I, the most advantageous metabolites in all species were amino acids and derivatives. According to the numbers of amino acids and derivatives among advantageous metabolites, the species were ranked in the following order: Gz (98) > Gl (71) > Gt (46) > Gs (15). Combining the results of Section 3.1 with these findings on dominant metabolites, it can be concluded that Gz is the most suitable among all *Ganoderma* species for developing amino acid products. Lipids were also found to be abundant in *Ganoderma*. According to the numbers of lipids, the species were ranked as follows: Gl (35) > Gt (33) > Gs (4) > Gz (1). The major lipids in Gl were comprised lysophosphatidylethanolamine and lysophosphatidylcholine, whereas those in Gt were mainly free fatty acids. Some fatty acids are essential because they cannot be synthesized by the human body. For instance, α-linolenic acid identified in this study has antithrombotic functions and is associated with reduced mortality rates of cardiovascular diseases and cancer (34, 35).

Terpenoids are considered one of the most important chemical compounds in *Ganoderma* (26, 36). According to the numbers of terpenoids among advantageous metabolites, the species were ranked as follows: Gs (9) = Gz (9) > Gt (2) > Gl (0). The contents of many terpenoids (91 in total) were relatively similar among all *Ganoderma* species; however, Gs and Gz were more abundant in advantageous terpenoids than the other species. Nearly all terpenoids found in *Ganoderma* have been demonstrated to possess beneficial physiological activities, such as anticancer and antioxidant properties (26, 36, 37). However, when we attempted to extract a large quantity of a specific terpenoid, we were unable to determine which species contains the highest amount and allows the highest extraction efficiency. Therefore, we presented the advantageous terpenoids in Supplementary Table S2 to facilitate better utilization of the terpenoids from each species.

Alkaloids are important chemical components of *Ganoderma*. Several studies have demonstrated that alkaloids in various *Ganoderma* products have remarkable health-promoting functions, including neuroprotection, renal protection, and anticancer effects (29, 38, 39). In total, 119 different alkaloids were identified in the four *Ganoderma* species, which calls for further research and development. According to the numbers of advantageous alkaloids among advantageous metabolites, the species were ranked as follows: Gl (20) > Gt (19) > Gz (5) > Gs (2). According to the pie chart in Supplementary Figure S2, Gl and Gt are more abundant in alkaloids than the other species. Compared to Gl, Gt has a wider variety of advantageous alkaloids. In addition to plumerane, which is also present in Gl, Gt contains pyridine alkaloids, pyrrole alkaloids, quinoline alkaloids, and piperidine alkaloids, which are absent in Gl. The alkaloids among the advantageous metabolites of all *Ganoderma* species are listed in Supplementary Table S2. *N*-Oleoylethanolamine exhibits weight-reducing and anti-atherosclerotic effects and is employed for the treatment of cardiovascular and metabolic disorders (40–42). *O*-Acetyl-l-carnitine functions in neuroprotection and the protection of brain development (43–45). Agmatine contributes to the regulation of glucose and lipid metabolism and has antidepressant, anticonvulsant, and neuroprotective effects (46, 47).

Nucleotides and derivatives are another important component of *Ganoderma* and derived health products; they exhibit pharmacological activities and health-promoting functions such as lipid-lowering effects (48–51). We identified 84 nucleotides and derivatives in the four *Ganoderma* species. According to the numbers of nucleotides and derivatives among advantageous metabolites, the species were ranked as follows: Gt (17) > Gl (14) > Gz (2) > Gs (0). The nucleotides and derivatives among advantageous metabolites of all *Ganoderma* species are listed in Supplementary Table S2. Vidarabine exhibits potent antiviral activity and is widely used as an anti-herpesvirus agent (52). Citicoline exerts neuroprotective effects and has anticonvulsant properties (53, 54). β-Nicotinamide mononucleotide possesses anti-aging properties (55, 56). Acadesine possesses antitumor activities in several cancer types (57, 58).

Flavonoids, phenolic acids, and organic acids among advantageous metabolites of four *Ganoderma* species are listed in Supplementary Table S2. Flavonoids are a research focus in *Ganoderma* studies and exhibit antioxidant and anti-inflammatory effects and cytotoxicity against cancer cells (59–62). Xanthohumol possesses antitumor activities and antiarrhythmic properties (63, 64). 2′,7-Dihydroxy-3′,4′-dimethoxyisoflavan possesses anti-inflammatory activity (65). The organic acid γ-aminobutyric acid improves sleep and reduces blood pressure (66–68). Shikimic acid has diverse biological activities and serves as an intermediate in the synthesis of anticancer drugs (69). The phenolic acids protocatechualdehyde, (E)-ethyl p-methoxycinnamate, and picein exhibit anticancer, anti-inflammatory, and antioxidant effects (70–73).

### 3.5 Network pharmacology analysis

#### 3.5.1 PPI networks of key targets from the four *Ganoderma* species

**Figure 7:**
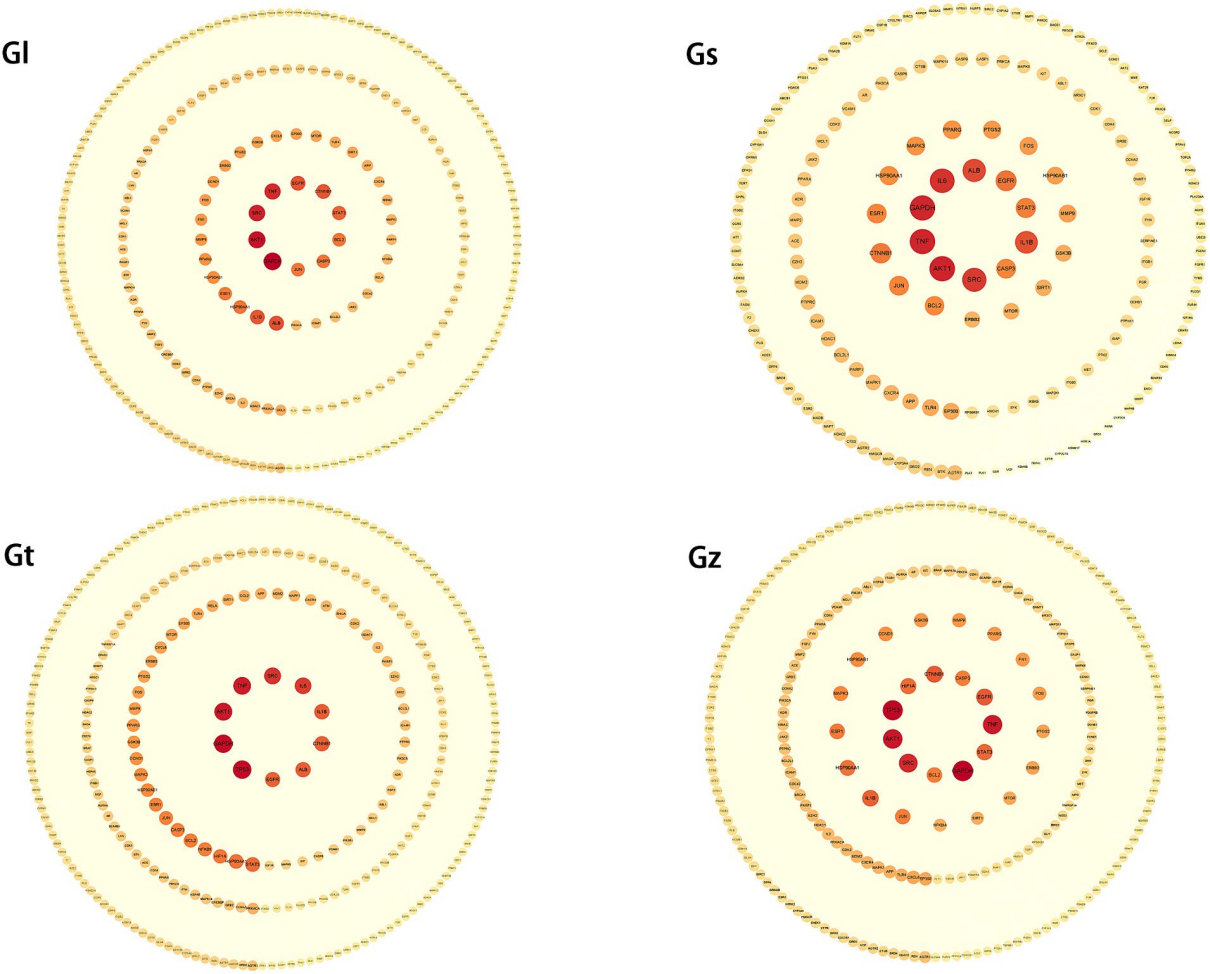
PPI networks of key targets from four *Ganoderma* species. The degree of the target increases proportionally with the darkness of the color and the size of the font.

PPI networks of key targets were constructed for advantageous metabolites of all *Ganoderma* species (Figure 7 and Supplementary Table S3). In each PPI network, SRC, GAPDH, TNF, and AKT1 were among the top five highest-degree targets. SRC is associated with increased tumor progression, invasion, and metastasis in many cancers (74, 75). TNF plays a crucial role in cancer treatment and autoimmunity (76, 77), while AKT1 is linked to breast cancer and ovarian cancer (78, 79). Despite differences in the advantageous metabolites from the four *Ganoderma* species, their mechanisms of tumor suppression are closely related to these targets.

The third highest-degree target of TP53 was identified in Gz and Gt, but not in Gl and Gs. TP53 functions as a crucial tumor suppressor, with its mutations being frequently observed in various malignant neoplasms (80, 81). This observation implies that the tumor-suppressive mechanisms of metabolites in Gz and Gt may exhibit greater prominence in regulating TP53 than those of metabolites in Gl and Gs. Albumin (ALB) exhibited a high degree in the PPI networks of Gl, Gs, and Gt. ALB is primarily synthesized by the liver in humans and serves as the predominant plasma protein (82). Extensive research has demonstrated that ALB functions as a tumor suppressor and plays a crucial role in hepatocellular carcinoma metastasis and invasion (83).

#### 3.5.2 KEGG pathway and GO annotation analyses

**Figure 8:**
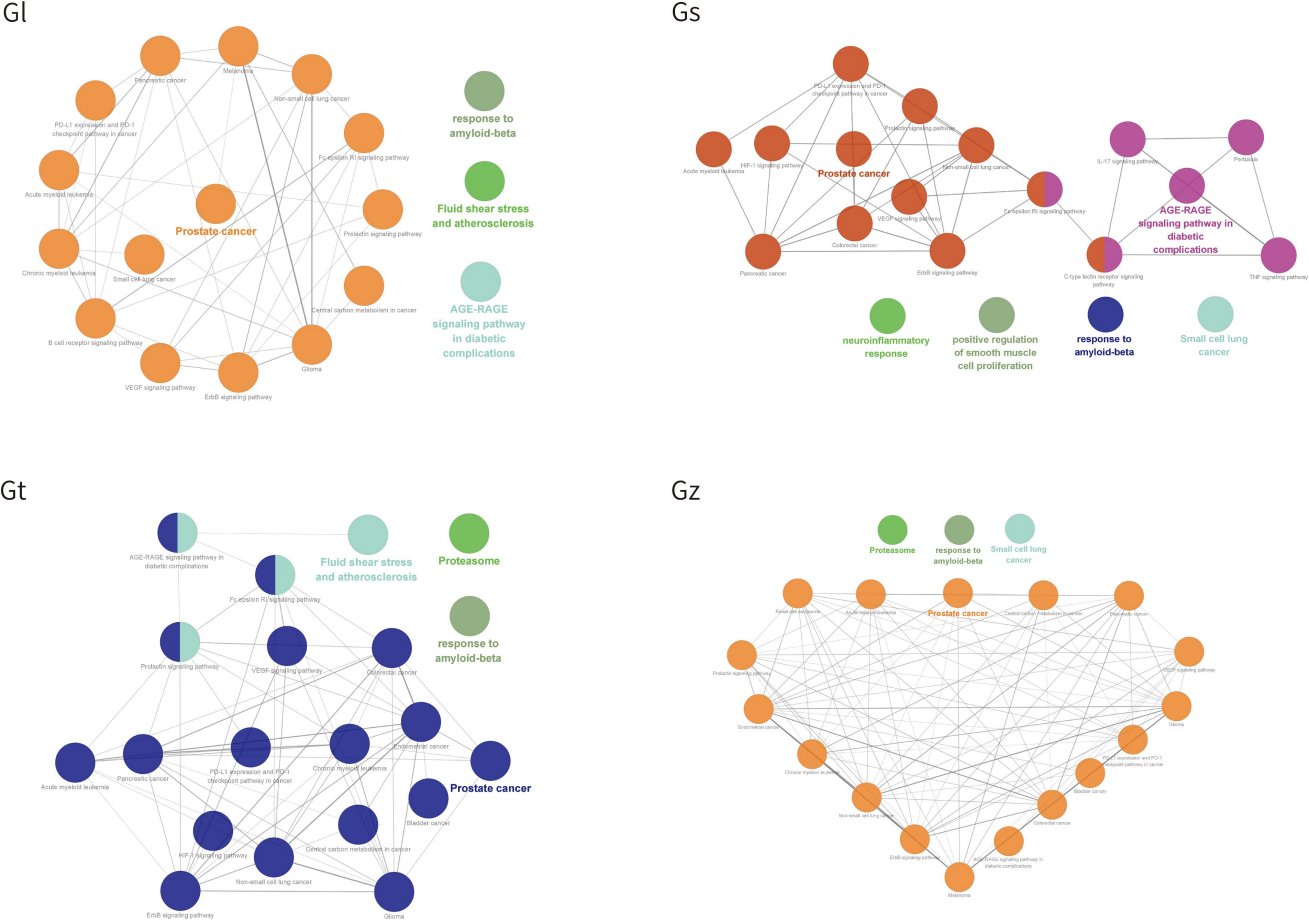
The top 20 pathway annotations in the ratio are presented by ClueGO.

GO and KEGG pathways were enriched and visualized by Cluego software (Figure 8). Among the top 20 pathways identified in the four *Ganoderma* species, several cancer-related pathways were prominently observed, including prostate cancer, non-small cell lung cancer, colorectal cancer, small cell lung cancer, pancreatic cancer, bladder cancer, and endometrial cancer. The advantageous metabolites of the four *Ganoderma* species exhibited a strong correlation with diverse cancer pathways, underscoring their potential as promising therapeutic agents. We also observed that some of the top 20 pathways were only found in certain species of *Ganoderma*, including cocaine addiction and B cell receptor signaling pathway in Gl; TNF signaling pathway, positive regulation of smooth muscle cell proliferation, IL-17 signaling pathway, C-type lectin receptor signaling pathway, pertussis, and neuroinflammatory response in Gs; and renal cell carcinoma in Gz. The findings facilitate a more precise investigation into the therapeutic pathways of different *Ganoderma* species.

**Figure 9:**
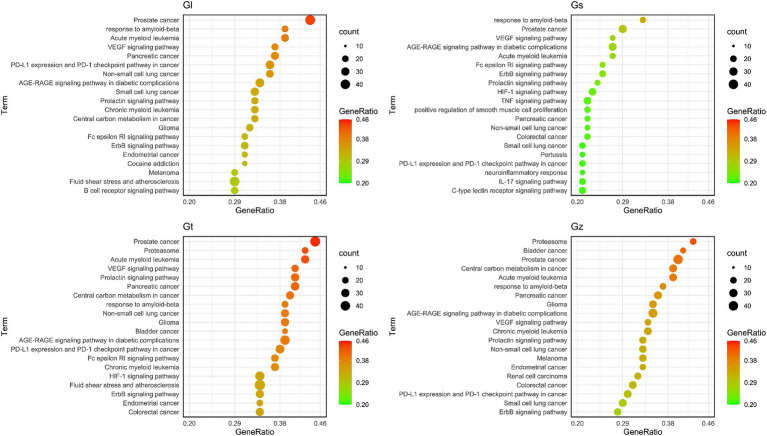
The bubble plot of the top 20 pathway from four *Ganoderma*.

The bubble plot was utilized to compare the number and ratio of genes in the pathways (Figure 9). Prostate cancer accounted for the highest ratio in all Gl and Gt pathways, reaching more than 44%. The proteasome exhibited a high ratio in both the Gz and Gt pathways. The response to amyloid-beta in the top 20 pathways of the four *Ganoderma* species exhibited a relatively high ratio. The neurotoxicity of amyloid-beta and its strong correlation with Alzheimer’s disease have been consistently demonstrated in recent studies (84, 85). Therefore, the neuroprotective effects of the four *Ganoderma* species may primarily be attributed to their modulation of the amyloid-beta pathway. Similar findings have also been corroborated in pharmacological analyses and validation studies involving *G. lucidum* (86, 87). The AGE-RAGE signaling pathway in diabetic complications had a high ratio in the four *Ganoderma* species. AGE-RAGE plays a crucial role in the development of diabetic complications, such as kidney disease and cardiovascular disease (88). Recent advancements in network pharmacological analysis and animal experiments have demonstrated that certain natural remedies, such as *Radix Rehmanniae* and *Corni Fructus*, can effectively regulate this pathway to combat diabetic complications (89); however, limited research has been conducted on *Ganoderma*.

## 4 Conclusion

We conducted a widely targeted metabolomics analysis of four *Ganoderma* species commonly used in the food and medical industries. The results indicate that although there are differences in the variety of metabolites among the four *Ganoderma* species, these differences are relatively small. However, there are significant differences in the content of metabolites among the four *Ganoderma* species. The relative amounts of many metabolites in different species of *Ganoderma* vary significantly by hundreds or even thousands of times. Therefore, even if the metabolite compositions of these four species of *Ganoderma* are similar, it is imperative to determine the difference in dosage when using them interchangeably. To achieve a more targeted application of *Ganoderma* in the medical and food fields and to facilitate further development and research of each *Ganoderma* species, we identified and discussed advantageous metabolites that are significantly more abundant in one *Ganoderma* species than in the others. Among the four *Ganoderma* species, Gz is the most suitable for the development of amino acid-based products. Gs and Gz are richer in terpenes, whereas Gl and Gt are more abundant in nucleotides and derivatives, alkaloids, and lipids than the other species. Network pharmacological analysis showed that the top 5 targets with high degree were similar, although the compositions of dominant metabolites in the four *Ganoderma* species were different. Simultaneously, certain discrepancies were also observed. For instance, among the highly correlated targets, TP53 was exclusively present in Gz and Gt, while ALB appeared in only Gl, Gs, and Gt. These variations may reflect the specificity of different *Ganoderma* species in targeting disease-related pathways. Furthermore, KEGG and GO analyses demonstrated that the advantageous metabolites of the four *Ganoderma* species have potential regulatory effects on various pathways associated with cancer, Alzheimer’s disease, and diabetes complications, among others. However, each *Ganoderma* species also displayed unique and significantly enriched pathways. The findings necessitate further comprehensive exploration and validation to facilitate the targeted utilization of diverse metabolites from *Ganoderma*. In the future, we should determine the absolute content of the advantageous metabolites of the four types of *Ganoderma* to determine their utilization value. At the same time, we should also study the thermal stability of the active ingredients in *Ganoderma* and explore the temperature range required to maintain their biological activity in daily processing.

## Data availability statement

The original contributions presented in the study are included in the article/Supplementary material, further inquiries can be directed to the corresponding author.

## Author contributions

LW-Y: Data curation, Formal analysis, Methodology, Software, Visualization, Writing – original draft, Writing – review & editing. GH-B: Conceptualization, Investigation, Supervision, Writing – original draft, Writing – review & editing, Formal analysis, Validation. YR-H: Investigation, Writing – review & editing. XA-G: Investigation, Writing – review & editing, Resources. ZJ-C: Visualization, Writing – original draft. YZ-Q: Writing – original draft, Resources. HW-J: Resources, Writing – original draft, Visualization. YX-D: Resources, Writing – original draft, Conceptualization, Funding acquisition, Investigation, Project administration, Supervision, Writing – review & editing.

## Footnotes

^1^ www.r-project.org
