# Supplementary material for: UPLC-ESI-MS/MS-based widely targeted metabolomics reveals differences in metabolite composition among four Ganoderma species
Source: Front Nutr. 2024 Mar 18;11:1335538. doi: 10.3389/fnut.2024.1335538 (PMC10982346; doi:10.3389/fnut.2024.1335538)
Supplement: Supplementary file 2 [file Data_Sheet_1.docx]

Supplementary Material

## Supplementary Figure

**
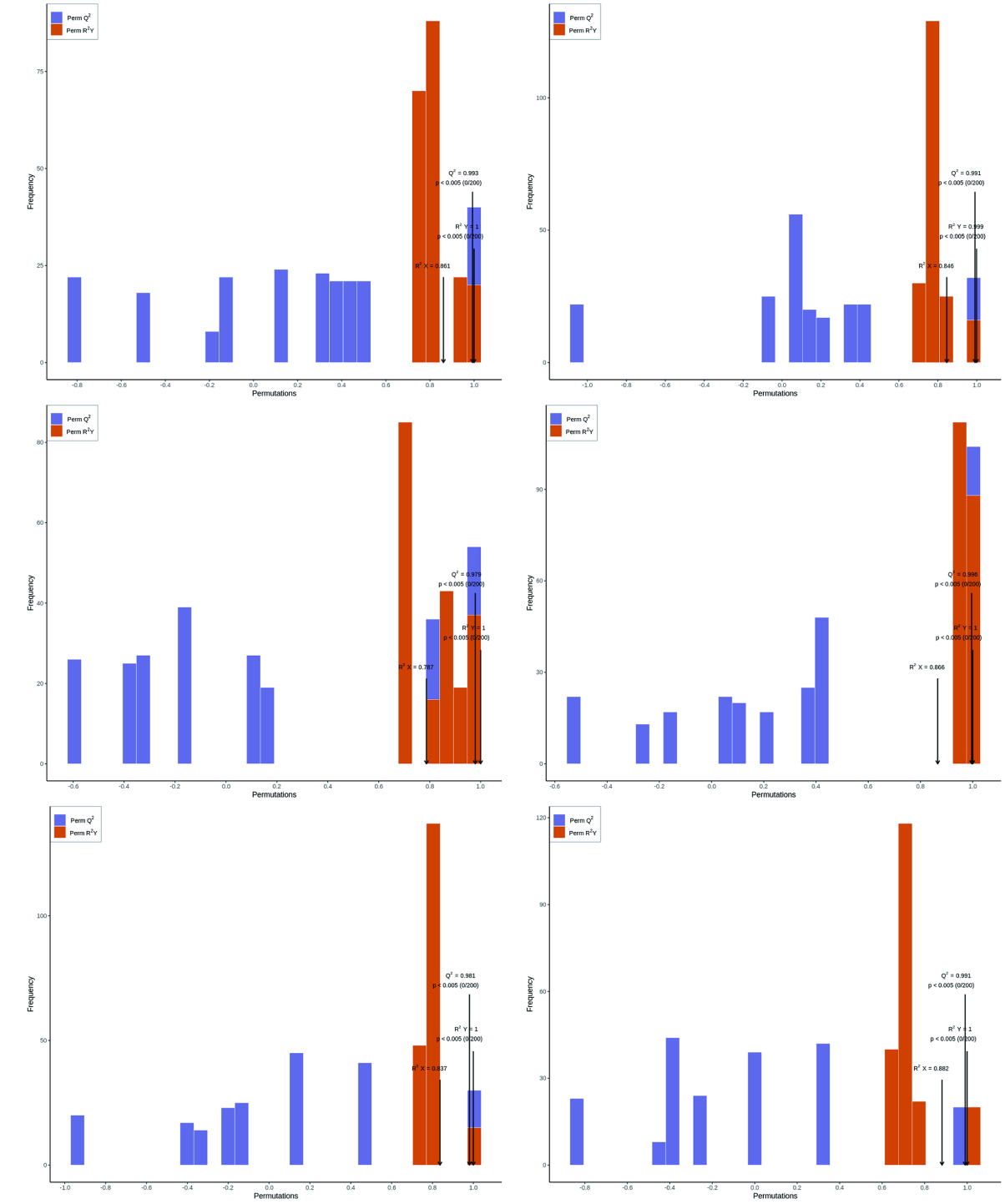
**

**Supplementary Figure 1 |** Verification diagrams of the OPLS-DA model for the four *Ganoderma* species. **(A)** Gl vs. Gs; **(B)** Gl vs. Gt; **(C)** Gl vs. Gz; **(D)** Gs vs. Gt; **(E)** Gz vs. Gs; **(F)** Gz vs. Gs; **(G)** Gz vs. Gt.

## Supplementary Tables

**Supplementary Table 1.** Details of the advantageous metabolites with FC >1,000. The compounds shared by both species are highlighted in orange, while those exclusive to one species are shown in a blank background.

|  | Compounds | Class I | Fold Change |
| --- | --- | --- | --- |
| Gs vs. Gl | Ser-Gly-Pro-Glu-Arg | Amino acids and derivatives | 2315518.89 |
|  | 4-Pyridoxic acid | Others | 661469.60 |
|  | Gln-Ala-His-Asp | Amino acids and derivatives | 500563.78 |
|  | Phe-Ala-Asn-Lys | Amino acids and derivatives | 307718.69 |
|  | Isobutyryl carnitine | Alkaloids | 169642.86 |
|  | NADP (Nicotinamide adenine dinucleotide phosphate) | Nucleotides and derivatives | 22327.60 |
|  | 1-(2,3-dihydroxypropoxy)-3-(((2-(dimethylamino)ethoxy)(hydroxy)phosphoryl)oxy)propan-2-yl (E)-hexadec-9-enoate | Lipids | 12736.60 |
|  | ATP; Adenosine 5'-Triphosphate | Nucleotides and derivatives | 11912.68 |
|  | 2-(2,3-dihydroxypropoxy)-3-(((2-(dimethylamino)ethoxy)(hydroxy)phosphoryl)oxy)propyl (8E,11Z,14Z)-octadeca-8,11,14-trienoate | Lipids | 4596.35 |
|  | Nicotinic acid adenine dinucleotide | Nucleotides and derivatives | 2007.82 |
|  | Val-Val | Amino acids and derivatives | 1771.53 |
|  | Phenylacetyl-L-glutamine | Amino acids and derivatives | 1417.55 |
|  | Met-Glu-Ser | Amino acids and derivatives | 1081.67 |
|  | Ser-Gln-Asp-Glu | Amino acids and derivatives | 1045.72 |
| Gt vs. Gl | LysoPC 19:0 | Lipids | 287693.09 |
|  | Ser-Gly-Pro-Glu-Arg | Amino acids and derivatives | 1342.70 |
| Gz vs. Gl | Phe-Ala-Asn-Lys | Amino acids and derivatives | 307718.69 |
|  | Isobutyryl carnitine | Alkaloids | 169642.86 |
|  | 5-Hydroxyferulate | Others | 130674.92 |
|  | Asn-Thr-Phe-Lys | Amino acids and derivatives | 88340.50 |
|  | 2-(2,3-dihydroxypropoxy)-3-(((2-(dimethylamino)ethoxy)(hydroxy)phosphoryl)oxy)propyl (8E,11Z,14Z)-octadeca-8,11,14-trienoate | Lipids | 4596.35 |
|  | Val-Val | Amino acids and derivatives | 3420.99 |
|  | LysoPC 20:2 | Lipids | 1150.87 |
| Gl vs. Gs | Ganoderic Acid Theta | Terpenoids | 5796.84 |
|  | Ganoderenic acid K | Terpenoids | 4128.71 |
|  | Ganoderic Acid M | Terpenoids | 1647.72 |
| Gt vs. Gs | LysoPE 16:3 | Lipids | 58060.36 |
|  | Ganoderenic acid K | Terpenoids | 2737.36 |
| Gz vs. Gs | 16-Hydroxy-24-methylene-3-oxolanosta-7,9(11)-diene-21-oic acid (Polyporenic acid C) | Terpenoids | 2546.55 |
| Gl vs. Gt | 9-Oxo-10E,12Z-octadecadienoic acid | Lipids | 65184.60 |
| Gs vs. Gt | Asn-Ser-Ala | Amino acids and derivatives | 270356.61 |
|  | Mevalonic acid | Organic acids | 66156.78 |
|  | p-Coumaroylferuloylcadaverine | Alkaloids | 35479.26 |
| Gz vs. Gt | N-Palmitoylglycine | Amino acids and derivatives | 14306.44 |
|  | 5-Methylcytidine | Nucleotides and derivatives | 1055.27 |
| Gl vs. Gz | Phe-Ala-Asp-Lys | Amino acids and derivatives | 5686537.68 |
|  | Ganoderic Acid GS-3 | Terpenoids | 453145.81 |
|  | Pro-Ile-Met | Amino acids and derivatives | 271867.16 |
|  | Glycyl-leucyl-tyrosine | Amino acids and derivatives | 143040.11 |
|  | 1-Methylguanidine | Alkaloids | 64931.57 |
|  | Nomilin | Terpenoids | 15615.33 |
| Gs vs. Gz | Trp-His-Ile | Amino acids and derivatives | 126582.97 |
|  | Nicotianamine | Alkaloids | 41090.58 |
|  | Phe-Ala-Asp-Lys | Amino acids and derivatives | 2008.80 |
| Gt vs. Gz | 2-[2-[(2-Amino-3-phenylpropanoyl)amino]propanoylamino]-3-hydroxypropanoic acid | Amino acids and derivatives | 783938.85 |
|  | Ganoderic Acid GS-3 | Terpenoids | 453145.81 |
|  | Gly-Thr-Gly | Amino acids and derivatives | 209104.28 |
|  | Val-Leu-Tyr | Amino acids and derivatives | 164256.81 |
|  | Phe-Ala-Leu | Amino acids and derivatives | 154422.82 |
|  | Trp-His-Ile | Amino acids and derivatives | 126582.97 |
|  | Nomilin | Terpenoids | 15615.33 |
|  | Phe-Ala-Asp-Lys | Amino acids and derivatives | 2805.95 |

**Supplementary Table 2.** Advantageous metabolites among four *Ganoderma* classified by substance category.

| Class Ⅰ | Class Ⅱ | Names of advantageous metabolites | Species |
| --- | --- | --- | --- |
| Terpenes | Triterpene | Ganoderic Acid Theta | 1. *sinense* |
|  |  | Kaneric acid |  |
|  |  | Polyporenic acid C |  |
|  |  | 6-Hydroxytrametenolic acid |  |
|  |  | Ganoderic Acid M |  |
|  |  | Camaldulenic acid |  |
|  |  | Ganoderic Acid I |  |
|  |  | Ganoderenic acid K |  |
|  |  | Lucidenic Acid C6 |  |
|  |  | Ganoderic acid L | *G. tsugae* |
|  |  | Rutundic acid |  |
|  |  | Lucidenic Acid F | *G. lingzhi* |
|  |  | Elfvingic acid A |  |
|  |  | Norarjunolic acid |  |
|  |  | Ganoderic Acid GS-3 |  |
|  |  | Lucidenic acid B |  |
|  |  | Uncargenin A |  |
|  |  | Ganoderenic Acid AM1 |  |
|  |  | Nomilin |  |
|  | Monoterpenoids | 12,13-Dehydrogeranylgeraniol |  |
| Alkaloids | Alkaloids | 2-(Acetylamino)-3-phenyl-2-propenoic acid | *G. leucocontextum* |
|  |  | 3-amino-2-naphthoic acid |  |
|  |  | Agmatine |  |
|  |  | Cadaverine |  |
|  |  | Isobutyryl carnitine |  |
|  |  | L-Tyramine |  |
|  |  | N-benzylformamide |  |
|  |  | O-Acetyl-L-carnitine |  |
|  |  | Spermidine |  |
|  |  | Valerine |  |
|  | Phenolamine | N-(2-Hydroxy-4-methoxyphenyl)acetamide |  |
|  |  | N-(3-hydroxy-4-methoxyphenethyl)-4-hydroxybutanamide |  |
|  |  | p-Aminoanisole |  |
|  | Piperidine alkaloids | N-ethylcytisine |  |
|  | Plumerane | 3-Hydroxy-3-methyloxindole |  |
|  |  | 3-Indole acetamide |  |
|  |  | 3-Indoleacrylic acid |  |
|  |  | Acetryptine |  |
|  |  | Indole-3-lactic acid |  |
|  |  | Tryptamine |  |
|  | Alkaloids | Deoxymutaaspergillic acid | *G. sinense* |
|  |  | 1-Methylhistamine |  |
|  |  | N-benzoyl-2-aminoethyl-β-D-glucopyranoside | *G. tsugae* |
|  |  | N-Oleoylethanolamine |  |
|  |  | Imidazol-1-yl-acetic acid |  |
|  |  | Zarzissine |  |
|  |  | Benzoyleneurea |  |
|  | Plumerane | Ailanindole |  |
|  |  | Indole-3-carboxaldehyde |  |
|  |  | Indole-5-carboxylic acid* |  |
|  | Phenolamine | 3-Hydroxyanthranilic acid |  |
|  |  | Dopamine |  |
|  |  | p-Coumaroylferuloylcadaverine |  |
|  |  | 4-Aminophenol |  |
|  |  | N-p-Coumaroylhydroxyagmatine |  |
|  |  | Vanillylamine |  |
|  | Piperidine alkaloids | 6-Deoxyfagomine |  |
|  | Pyridine alkaloids | 3-Succinoylpyridine |  |
|  |  | Nicotine |  |
|  | Pyrrole alkaloids | Pterolactam |  |
|  | Quinoline alkaloids | 2,4-Dihydroxyquinoline |  |
|  | Alkaloids | 1-Methylguanidine | *G. lingzhi* |
|  |  | Nicotianamine |  |
|  |  | Aurantiamide acetate |  |
|  | Phenolamine | Feruloylspermidine |  |
|  |  | Salicylamide |  |
| Nucleotides and derivatives | Nucleotides and derivatives | Vidarabine | *G. leucocontextum* |
|  |  | Adenosine |  |
|  |  | Inosine 5'-monophosphate |  |
|  |  | Adenosine 5'-diphosphate |  |
|  |  | Nicotinic acid adenine dinucleotide |  |
|  |  | Adenosine 5'-monophosphate |  |
|  |  | Uridine 5'-diphospho-D-glucose |  |
|  |  | Citicoline |  |
|  |  | Uridine-5'-diphospho-N-acetylgalactosamine disodium salt |  |
|  |  | 3'-Adenylic Acid |  |
|  |  | β-Nicotinamide mononucleotide |  |
|  |  | Uridine-5'-diphosphoglucuronic acid |  |
|  |  | NADP (Nicotinamide adenine dinucleotide phosphate) |  |
|  |  | ATP; Adenosine 5'-Triphosphate |  |
|  |  | 5-Methylcytidine | *G. tsugae* |
|  |  | L-Sepiapterin |  |
|  |  | N6-methyladenosine |  |
|  |  | N6-Isopentenyladenine |  |
|  |  | 2'-Deoxyuridine |  |
|  |  | N-(1-Deoxy-1-fructosyl)Valine |  |
|  |  | 2'-O-Methyladenosine |  |
|  |  | 3-Methyluridine |  |
|  |  | Xanthine |  |
|  |  | N-(1-Deoxy-1-fructosyl)Tryptophan |  |
|  |  | 8-Azaguanine |  |
|  |  | AICAR phosphate (Acadesine phosphate) |  |
|  |  | 2-Deoxyribose-5'-phosphate |  |
|  |  | N6-(2-Hydroxyethyl)adenosine |  |
|  |  | 1-Methylxanthine |  |
|  |  | 2-(Dimethylamino)guanosine |  |
|  |  | 7-Methylguanine |  |
|  |  | n6-(cis-hydroxyisopentenyl)adenosine | *G. lingzhi* |
|  |  | 8-Hydroxyguanosine |  |
|  |  | 2'-Deoxyguanosine |  |
| Flavonoids | Chalcones | Xanthohumol | *G. tsugae* |
|  | Other Flavonoids | 2',7-Dihydroxy-3',4'-dimethoxyisoflavan |  |
|  | Chalcones | 2,4,4'-trihydroxydihydrochalcone | *G. lingzhi* |
|  | Flavanonols | Aromadendrin (Dihydrokaempferol) |  |
| Phenolic acids | Phenolic acids | Benzamide | *G. leucocontextum* |
|  |  | 3-hydroxybenzaldehyde |  |
|  |  | Methyl 2,4-dihydroxyphenylacetate |  |
|  |  | (S)-2-Hydroxy-3-(4-Hydroxyphenyl)Propanoic Acid |  |
|  |  | 4-Hydroxyphenyllactic Acid |  |
|  |  | 2,4,6-trihydroxybenzaldehyde | *G.sinense* |
|  |  | Benzoylmalic acid |  |
|  |  | 2,5-Dihydroxyacetophenone |  |
|  |  | 2',4'-Dihydroxyacetophenone |  |
|  |  | Phenol |  |
|  |  | Salicylic acid | *G. tsugae* |
|  |  | 3,4'-Dihydroxy-3',5'-dimethoxypropiophenone |  |
|  |  | (E)-Ethyl p-methoxycinnamate |  |
|  |  | 3,4-Dihydroxybenzeneacetic acid |  |
|  |  | 3-Aminosalicylic acid |  |
|  |  | Tyrosol; 4-Hydroxyphenylethanol |  |
|  |  | 4-Hydroxybenzoic acid |  |
|  |  | 3-Methylsalicylic Acid |  |
|  |  | Protocatechualdehyde |  |
|  |  | 3-Methoxybenzoic acid |  |
|  |  | Vanillin acetate |  |
|  |  | 3-hydroxy-5-methoxybenzaldehyde | *G. lingzhi* |
|  |  | Picein (4-Acetylphenyl-glucoside) |  |
|  |  | Maleoyl-caffeoylquinic acid |  |
| Organic acids | Organic acids | γ-Aminobutyric acid | *G. leucocontextum* |
|  |  | Adenylocuccinic Acid |  |
|  |  | Mandelic acid |  |
|  |  | 6-Hydroxyhexanoic acid |  |
|  |  | 2,2-Dimethylsuccinic acid |  |
|  |  | Tranexamic Acid |  |
|  |  | Argininosuccinic acid |  |
|  |  | phenylacetic acid |  |
|  |  | Succinic semialdehyde |  |
|  |  | Methanesulfonic acid | *G. sinense* |
|  |  | 3-Amino-1-propionic sulfonic acid |  |
|  |  | 2-Methyl-3-(pyrimidin-2-YL)propanoic acid | *G. tsugae* |
|  |  | 2-Hydroxymyristic acid |  |
|  |  | 2-Hydroxyhexadecanoic acid |  |
|  |  | 4-Oxatetradecanoic acid |  |
|  |  | Pyrrole-2-carboxylic acid |  |
|  |  | Sebacic acid |  |
|  |  | β-Hydroxyisovaleric acid |  |
|  |  | 4-Oxopentanoic Acid |  |
|  |  | Mevalonic acid |  |
|  |  | Aminomalonic acid |  |
|  |  | Ethylmalonic acid |  |
|  |  | Succinic acid |  |
|  |  | 4,8-Dihydroxyquinoline-2-carboxylic acid |  |
|  |  | Rotenonic acid |  |
|  |  | Suberic Acid |  |
|  |  | 4-Hydroxycrotonic acid |  |
|  |  | Shikimic acid |  |
|  |  | 3-Methyl-2-Oxobutanoic acid |  |
|  |  | 6-Aminocaproic acid |  |
|  |  | 1-Aminocyclopropane-1-carboxylic acid |  |
|  |  | DL-Glyceric Acid |  |
|  |  | D-Mandelic acid | *G. lingzhi* |
|  |  | Urocanic acid |  |
